# Empathy and theory of mind in multiple sclerosis: A protocol for systematic review and meta-analysis

**DOI:** 10.1097/MD.0000000000021773

**Published:** 2020-08-14

**Authors:** XiaoGuang Lin, XueLing Zhang, QinQin Liu, PanWen Zhao, JianGuo Zhong, PingLei Pan, GenDi Wang, ZhongQuan Yi

**Affiliations:** a Department of Neurology, Affiliated Suqian Hospital of Xuzhou Medical University, Suqian; b Department of Central Laboratory; c Department of Neurology, Affiliated Yancheng School of Clinical Medicine of Nanjing Medical University, P.R. China.

**Keywords:** empathy, meta-analysis, multiple sclerosis, protocol, systematic review, theory of mind

## Abstract

**Background:**

Multiple sclerosis (MS) is an immune-mediated demyelinating disease of the central nervous system (CNS). Recently, numerous studies have shown that MS disrupts a number of social cognitive abilities, including empathy, theory of mind (ToM), and facial emotion recognition. In contrast to well-documented deficits in the core social cognitive domains of ToM and facial emotion recognition, it is not clear the broad and specific subcomponents of empathy processing affected. In addition, the specific subcomponents of ToM affected in MS are also unclear. The aim of this study is to conduct a systematic review and meta-analysis to characterize the performance of empathy and ToM in MS.

**Methods:**

A systematic literature search will be performed for eligible studies published up to July 1st, 2020 in 3 international databases (PubMed, Web of Science, and Embase). The work such as article retrieval, screen, quality evaluation, data collection will be conducted by 2 independent researchers. Meta-analysis will be performed using Stata 15.0 software.

**Results:**

The results of this study will be published in a peer-reviewed journal.

**Conclusions:**

This meta-analysis will provide a high-quality synthesis from existing evidence for the performance of empathy and ToM in MS.

**PROSPERO registration number:**

INPLASY202070029.

## 1 Introduction

Multiple sclerosis (MS) is an immune-mediated demyelinating disease of the central nervous system (CNS),^[1]^ which is characterized by the occurrence of widespread lesions or plaques in the brain and spinal cord.^[2,3]^ Due to these unpredictable lesions, MS results in overburdening patients and extensive clinical manifestations, including muscle weakness, sensory deficits, fatigue, and cognitive impairment.^[4]^

In MS patients, cognitive impairment is common, that not only includes deficits in abilities assessed by traditional neuropsychological batteries, such as executive functioning, information processing speed, attention, and memory,^[5,6]^ but also often deficits in social cognition.^[7–11]^ Social cognition is a basic mean for people to perceive, process, and interpret social information, which has a drastic impact on interpersonal communication and quality of life.^[12–15]^ Social cognition is not a unitary skill, but a multidimensional construct that involves empathy, theory of mind (ToM), and facial emotion recognition.^[16]^

Empathy, one core domain of social cognitive, refers to the ability to understand and identify the mental states of others, as well as our ability to share the feelings of others.^[17]^ It is a multifaceted construct, including the cognitive and affective subcomponents of empathy. The cognitive empathic referring to the ability to understand what others’ are feeling, and the affective empathic describing one's emotional response to the perceived situation of another.^[18,19]^ These 2 aspects of empathy rely on different brain structures, and take different developmental pathways.^[18]^ This is significant in clinical practice, as any deficit in cognitive empathy or affective empathy can lead to atypical emotional reactions, but clinical treatment implications are different.^[17,20]^ To our knowledge, there has been no meta-analytic study to quantitatively test the magnitude and significance of any MS-related effects in empathy.

Like empathy, ToM is another core domain of social cognitive, which is the ability to attribute mental states to others, and to use the attributions to understand and predict behavior.^[21,22]^ For ToM, the affective and cognitive subcomponents could be identified by content of the stimuli used in ToM tasks.^[23]^ Cognitive ToM requires an understanding of another's thoughts, intentions, and beliefs, affective ToM is concerned with understanding what another is feeling.^[24]^ To our knowledge, 2 recent meta-analyses have found evidences of moderate sized ToM deficits in patients with MS.^[25,26]^ However, it was not clear whether these defects were attributable to both or only one subcomponent, as no specific analysis was conducted for cognitive ToM or affective ToM.

Notably, there are differences between affective ToM and cognitive empathy in definition,^[27]^ but these 2 constructs are difficult to be distinguished at a purely behavioral level of assessment, as they both involve an attribution of another's emotional state.^[28]^ Besides, in recent studies, the overlap between affective ToM and cognitive empathy has often been noted.^[20,29,30]^ Therefore, we consider that affective ToM and cognitive empathy are 2 interchangeable terms in this paper.

In sum, we will conduct a systematic review and meta-analysis to systematically characterize the performance of empathy and ToM in MS. The quantitative analysis will be conducted by incorporating both empathy and ToM as a broad construct. Besides, we will conduct specific analysis for the overlapping components (affective ToM and cognitive empathy) and separate components (affective empathy and cognitive ToM). In addition, we will evaluate potential moderators of impairments observed in these individuals to help explain any variability between studies. Our meta-analysis will be helpful to promote a more comprehensive and nuanced understanding of how these 2 core domains of social cognitive are affected in MS.

## 2 Methods

### 2.1 Study registration

This systematic review has been registered on INPLASY (INPLASY202070029, URL = https://inplasy.com/inplasy-2020-7-0029/), which was reported based on the guidelines of the Preferred Reporting Items for Systematic Reviews and Meta-Analyses Protocols (PRISMA-P) statement.^[31]^

### 2.2 Ethical approval

Ethical approval is not required because the data used in this paper are from published studies without the involvement of individual or animals experiments.

### 2.3 Criteria of selection for study

#### 2.3.1 Criteria for inclusion

Studies were considered eligible for inclusion if the study compared MS participants with a matched healthy controls group, the study had to assess empathy performance or ToM performance using standard measures, sufficient data to calculate effect sizes and standard errors of the empathy or ToM were reported, the study was published in a peer-reviewed journal in English. Studies were considered eligible for exclusion if the study with the patient samples was overlapped with another one with a larger sample size, the study lacked a healthy controls (HC) group, the study with a sample size under 10 will be excluded to ensure the reliability of the outcome,^[21]^ the publication was not an original type, such as research protocols, letters, conference abstracts, reviews, and editorials.

#### 2.3.2 Criteria for exclusion

Studies were considered eligible for exclusion if the study with the patient samples was overlapped with another one with a larger sample size, the study lacked an HC group, the study with a sample size under 10 will be excluded to ensure the reliability of the outcome,^[21]^ the publication was not an original type, such as research protocols, letters, conference abstracts, reviews, and editorials.

#### 2.3.3 Types of participants

Patients diagnosed with MS will be included in the study. Patients with other serious complications, a history of brain surgery, or other serious neurodegenerative diseases will be excluded from this study.

#### 2.3.4 Types of interventions

We will mainly study the performance of empathy and ToM between MS patients and healthy controls.

#### 2.3.5 Type of comparators

We will choose healthy controls.

#### 2.3.6 Types of outcome measures

Main results: the measures of empathy Science and Embase. The search is from inception to July 1st, 2020 with no restriction of publication dates. In addition, other resources will be searched manually, such as the references of all included studies.

### 2.4 Data sources

#### 2.4.1 Electronic searches

Three electronic databases (PubMed, Web of Science, and Embase) have to be searched from inception to July 1st, 2020. There were no restrictions of the age of patients or phenotype of MS for inclusion. In addition, other resources will be searched manually, such as the references of all included studies.

#### 2.4.2 Search strategy

Search terms are related to MS and empathy/ToM. Related Medical Subject Heading (MeSH) terms and synonyms in various combinations are used as search strategies. The terms to be used in relation to the disease include multiple sclerosis, MS, and clinically isolated syndrome. The terms to be used in relation to the empathy/ToM include social cognition, theory of mind, ToM, mentalizing, mentalizing, facial expression∗, prosody, pragmatic impairment, non-literal language, sarcas∗, lie∗, joke∗, empath∗, perspective taking, and Peer-Report Social Functioning Scale. The search strategy in the PubMed, Web of Science, and Embase databases are shown in Table 1.

**Table 1:**
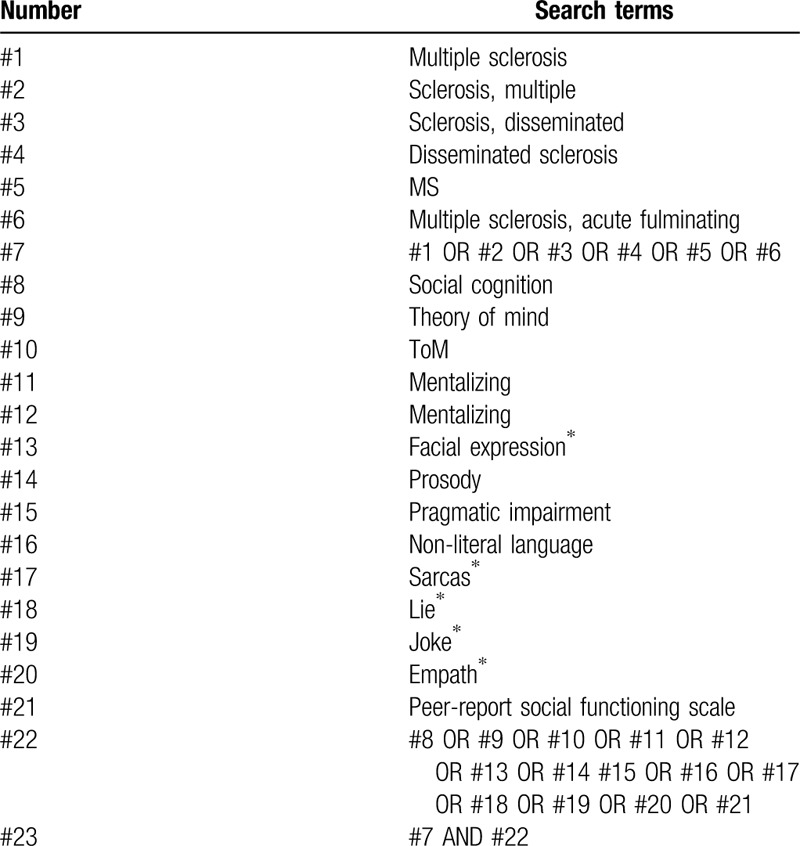
Represents the search strategy for PubMed database.

### 2.5 Data collection and analysis

#### 2.5.1 Selection of studies

**Figure 1:**
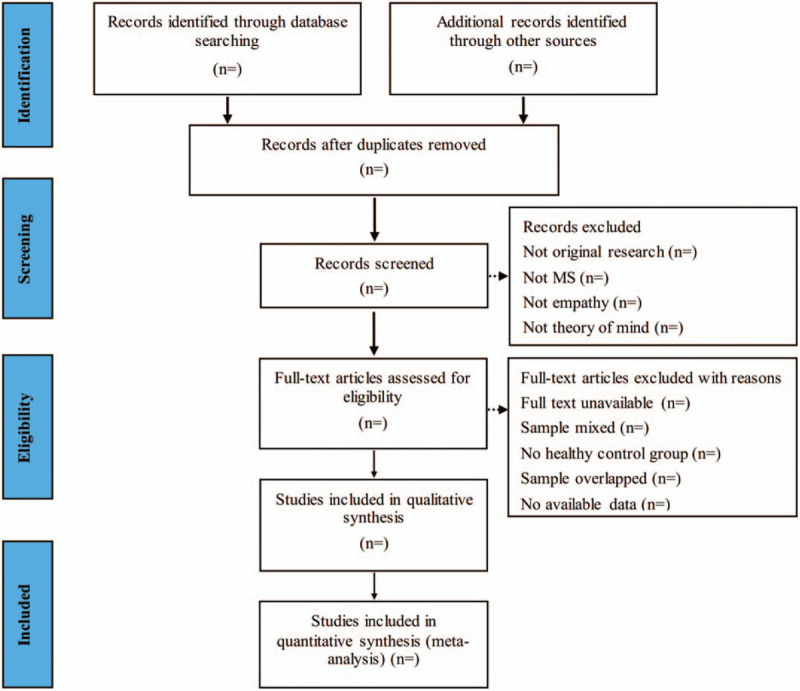
Flow diagram of studies search and selection.

The study selection process will be presented in the following PRISMA flow diagram (Fig. 1). We will manage all literatures by using EndNote software, V.X9 (United States). Two investigators will independently review and screen the literature based on predetermined inclusion and exclusion criteria. If there is a disagreement between the 2 investigators, we will discuss to solve it. If there are still objections, the third reviewer will analyze them. The reasons for the excluded articles will be recorded.

#### 2.5.2 Assessment of quality in included studies

We will use the Newcastle-Ottawa Quality Assessment Scale (NOS) to assess the quality of all included studies.^[32]^

#### 2.5.3 Data extraction and management

A unified data extraction form will be designed. Two investigators will independently extract data. The information will include first author, publication year and title, MS diagnosis criteria, inclusion/exclusion criteria, number of groups, number of participants, patients’ age, sex, education level, disease duration, healthy controls’ age, sex, education level, the measures of empathy/ToM, the data used for calculating the effect sizes and standard errors of the empathy/ToM measures. Any disagreement will be discussed between the 2 investigators, and further disagreements will be arbitrated by the third author.

### 2.6 Data synthesis and statistical analysis

#### 2.6.1 Measures of treatment effect

Stata 15.0 software (STATA Corp, College Station, TX) will be used for data analysis and quantitative data synthesis. The mean effect size (Hedge's *g*) and 95% confidence intervals (CI) will be used to evaluate the performance of empathy and ToM.^[33]^

#### 2.6.2 Dealing with missing data

For included studies in which there are missing data or the analysis process is unclear, the associated risk of bias will be fully considered. The authors will be contacted via email about information that is not available in the study. If data are still insufficient after contacting the author, it will be analyzed using the available data.

#### 2.6.3 Data synthesis

For studies reporting >1 ToM task, pooled effect size and standard error value were calculated.^[34]^ Effect sizes <0.5 were considered small, between 0.5 and 0.8 moderate, and >0.8 large.^[35]^ When appropriate, data will be pooled across studies for meta-analysis using fixed or random effect models.

#### 2.6.4 Assessment of heterogeneity

We will assess the heterogeneity by the *I*^2^ statistic base on a standard linear hypothesis with *I*^2^ < 50 indicating low heterogeneity.^[36]^ If *I*^2^ value is <50%, we will apply fixed-effects model to homogeneous data, otherwise the random-effects model will be applied.

#### 2.6.5 Assessment of publication bias

We will use funnel plots to detect publication bias. If the analysis includes ≥10 studies in meta-analysis, a test for funnel plot asymmetry using Egger method will be conducted.^[37]^

#### 2.6.6 Sensitivity analysis

We will conduct a sensitivity analysis to assess the reliability and robustness of the aggregation results via eliminating trials with high bias risk. If reporting bias was found, we will apply the trim-and-fill method to provide effect sizes adjusted for publication bias.^[38]^

#### 2.6.7 Subgroup analysis

If the heterogeneity of the results is high and the data are sufficient, we will perform a subgroup analysis on the data in order to find the cause of the large heterogeneity. Subgroup analysis will be performed according to clinical subtypes (such as clinically isolated syndrome, relapsing-remitting MS, progressive primary MS, and secondary progressive MS).

#### 2.6.8 Meta-regression analysis

Meta-regression analyses will be conducted for variables including the age, sex, education level, and disease duration, with a random-effects model using the restricted-information maximum likelihood method with the significance level set at *P* < .05.

## 3 Discussion

To our knowledge, this is the first research protocol to examine the performance of empathy and ToM in MS. In this systematic review and meta-analysis, the data will provide important clarifications about how MS affects the 2 core domains of social cognition. Now, it is well accepted that in many neurological groups, social cognitive impairment is a key predictor of broader prognostic outcomes, including mental health, social function, and quality of life. This meta-analysis will be helpful to promote a more comprehensive and nuanced understanding of how social cognitive is affected in MS. Social cognitive training has been shown to be effective in other disorders^[39]^ and it is hoped that our result can be helpful for informing the development of similar interventions for those with MS.

## Author contributions

**Conceptualization:** XiaoGuang Lin, XueLing Zhang.

**Data curation:** QinQin Liu, PanWen Zhao.

**Investigation:** XiaoGuang Lin, PanWen Zhao.

**Methodology:** PingLei Pan, JianGuo Zhong.

**Supervision:** PingLei Pan, JianGuo Zhong.

**Validation:** GenDi Wang.

**Writing – original draft:** XiaoGuang Lin, GenDi Wang.

**Writing – review & editing:** ZhongQuan Yi, GenDi Wang.

## References

[1] Multiple sclerosis. Nat Rev Dis Primers 2018;4:44. 30410088 10.1038/s41572-018-0046-z

[2] Buchanan RJ Minden SL Chakravorty BJ A pilot study of young adults with multiple sclerosis: demographic, disease, treatment, and psychosocial characteristics. Disabil Health J 2010;3:262–70. 21122795 10.1016/j.dhjo.2009.09.003

[3] Motl RW Sandroff BM Kwakkel G Exercise in patients with multiple sclerosis. Lancet Neurol 2017;16:848–56. 28920890 10.1016/S1474-4422(17)30281-8

[4] Henry A Tourbah A Chaunu MP Social cognition impairments in relapsing-remitting multiple sclerosis. J Int Neuropsychol Soc 2011;17:1122–31. 22014035 10.1017/S1355617711001147

[5] Jongen PJ Ter Horst AT Brands AM Cognitive impairment in multiple sclerosis. Minerva Med 2012;103:73–96. 22513513

[6] Miller E Morel A Redlicka J Pharmacological and non-pharmacological therapies of cognitive impairment in multiple sclerosis. Curr Neuropharmacol 2018;16:475–83. 29119933 10.2174/1570159X15666171109132650 PMC6018194

[7] Pitteri M Genova H Lengenfelder J Social cognition deficits and the role of amygdala in relapsing remitting multiple sclerosis patients without cognitive impairment. Mult Scler Relat Disord 2019;29:118–23. 30710839 10.1016/j.msard.2019.01.030

[8] Isernia S Cabinio M Pirastru A Theory of Mind network in Multiple Sclerosis: a double disconnection mechanism. Soc Neurosci 2020;1–4. 10.1080/17470919.2020.1766562 32378482

[9] Genova HM McDonald S Social cognition in individuals with progressive multiple sclerosis: a pilot study using TASIT-S. J Int Neuropsychol Soc 2020;26:539–44. 31948498 10.1017/S1355617719001371

[10] Neuhaus M Bagutti S Yaldizli Ö Characterization of social cognition impairment in multiple sclerosis. Eur J Neurol 2018;25:90–6. 28898535 10.1111/ene.13457

[11] Phillips LH Henry JD Scott C Specific impairments of emotion perception in multiple sclerosis. Neuropsychology 2011;25:131–6. 21090898 10.1037/a0020752

[12] Bora E Walterfang M Velakoulis D Theory of mind in Parkinson's disease: a meta-analysis. Behav Brain Res 2015;292:515–20. 26166188 10.1016/j.bbr.2015.07.012

[13] Green MF Penn DL Bentall R Social cognition in schizophrenia: an NIMH workshop on definitions, assessment, and research opportunities. Schizophr Bull 2008;34:1211–20. 18184635 10.1093/schbul/sbm145 PMC2632490

[14] Rao SM Leo GJ Ellington L Cognitive dysfunction in multiple sclerosis. II. Impact on employment and social functioning. Neurology 1991;41:692–6. 1823781 10.1212/wnl.41.5.692

[15] Krause M Wendt J Dressel A Prefrontal function associated with impaired emotion recognition in patients with multiple sclerosis. Behav Brain Res 2009;205:280–5. 19686782 10.1016/j.bbr.2009.08.009

[16] Green MF Horan WP Lee J Social cognition in schizophrenia. Nat Rev Neurosci 2015;16:620–31. 26373471 10.1038/nrn4005

[17] Henry JD von Hippel W Molenberghs P Clinical assessment of social cognitive function in neurological disorders. Nat Rev Neurol 2016;12:28–39. 26670297 10.1038/nrneurol.2015.229

[18] Bartochowski Z Gatla S Khoury R Empathy changes in neurocognitive disorders: a review. Ann Clin Psychiatry 2018;30:220–32. 30028897

[19] Wondra JD Ellsworth PC An appraisal theory of empathy and other vicarious emotional experiences. Psychol Rev 2015;122:411–28. 25961468 10.1037/a0039252

[20] Dvash J Shamay-Tsoory SG Theory of mind and empathy as multidimensional constructs. Top Lang Disord 2014;34:282–95.

[21] Leppanen J Sedgewick F Treasure J Differences in the Theory of Mind profiles of patients with anorexia nervosa and individuals on the autism spectrum: a meta-analytic review. Neurosci Biobehav Rev 2018;90:146–63. 29656033 10.1016/j.neubiorev.2018.04.009

[22] McDonald S Flanagan S Social perception deficits after traumatic brain injury: interaction between emotion recognition, mentalizing ability, and social communication. Neuropsychology 2004;18:572–9. 15291735 10.1037/0894-4105.18.3.572

[23] Yi Z Zhao P Zhang H Theory of mind in Alzheimer's disease and amnestic mild cognitive impairment: a meta-analysis. Neurol Sci 2020;41:1027–39. 31912336 10.1007/s10072-019-04215-5

[24] Heitz C Noblet V Phillipps C Cognitive and affective theory of mind in dementia with Lewy bodies and Alzheimer's disease. Alzheimers Res Ther 2016;8:10. 26979460 10.1186/s13195-016-0179-9 PMC4793654

[25] Bora E Özakbaş S Velakoulis D Social cognition in multiple sclerosis: a meta-analysis. Neuropsychol Rev 2016;26:160–72. 27324894 10.1007/s11065-016-9320-6

[26] Cotter J Firth J Enzinger C Social cognition in multiple sclerosis: a systematic review and meta-analysis. Neurology 2016;87:1727–36. 27655736 10.1212/WNL.0000000000003236 PMC5085073

[27] Singer T The neuronal basis and ontogeny of empathy and mind reading: review of literature and implications for future research. Neurosci Biobehav Rev 2006;30:855–63. 16904182 10.1016/j.neubiorev.2006.06.011

[28] Bensalah L Caillies S Anduze M Links among cognitive empathy, theory of mind, and affective perspective taking by young children. J Genet Psychol 2016;177:17–31. 26508454 10.1080/00221325.2015.1106438

[29] Preckel K Kanske P Singer T On the interaction of social affect and cognition: empathy, compassion and theory of mind. Curr Opin Behav Sci 2018;19:1–6.

[30] Shamay-Tsoory SG Aharon-Peretz J Perry D Two systems for empathy: a double dissociation between emotional and cognitive empathy in inferior frontal gyrus versus ventromedial prefrontal lesions. Brain 2009;132:617–27. 18971202 10.1093/brain/awn279

[31] Gharahkhani P Fitzgerald RC Vaughan TL Genome-wide association studies in oesophageal adenocarcinoma and Barrett's oesophagus: a large-scale meta-analysis. Lancet Oncol 2016;17:1363–73. 27527254 10.1016/S1470-2045(16)30240-6 PMC5052458

[32] Stang A Critical evaluation of the Newcastle-Ottawa scale for the assessment of the quality of nonrandomized studies in meta-analyses. Eur J Epidemiol 2010;25:603–5. 20652370 10.1007/s10654-010-9491-z

[33] Hedges LV Distribution theory for Glass's estimator of effect size and related estimators. J Educ Stat 1981;6:107–28.

[34] Scammacca N Roberts G Stuebing KK Meta-analysis with complex research designs: dealing with dependence from multiple measures and multiple group comparisons. Rev Educ Res 2014;84:328–64. 25309002 10.3102/0034654313500826 PMC4191743

[35] Routledge Academic, Cohen J Statistical Power Analysis for the Behavioral Sciences. 2013.

[36] Higgins JP Thompson SG Quantifying heterogeneity in a meta-analysis. Stat Med 2002;21:1539–58. 12111919 10.1002/sim.1186

[37] Egger M Smith GD Schneider M Bias in meta-analysis detected by a simple, graphical test. BMJ 1997;315:629–34. 9310563 10.1136/bmj.315.7109.629 PMC2127453

[38] Duval S Tweedie R Trim and fill: a simple funnel-plot-based method of testing and adjusting for publication bias in meta-analysis. Biometrics 2000;56:455–63. 10877304 10.1111/j.0006-341x.2000.00455.x

[39] Kurtz MM Gagen E Rocha NB Comprehensive treatments for social cognitive deficits in schizophrenia: a critical review and effect-size analysis of controlled studies. Clin Psychol Rev 2016;43:80–9. 26437567 10.1016/j.cpr.2015.09.003

